# Is there a reason for performing frontal sinus trephination at 1 cm from midline? A tomographic study.

**DOI:** 10.1016/S1808-8694(15)30996-4

**Published:** 2015-10-19

**Authors:** Otavio Bejzman Piltcher, Marcelo Antunes, Fernanda Monteiro, Cláudia Schweiger, Barry Schatkin

**Affiliations:** a MS. PhD, Assistant Physician; b Otorhinolaryngologist, Former resident – Porto Alegre University Hospital; c Otorhinolaryngologist, Former resident – Porto Alegre University Hospital; d MD. Otorhinolaryngologist Resident – Porto Alegre University Hospital; e MS. PhD, Otorhinolaryngology Professor, University of Pittsburgh. Otorhinolaryngologist Department – Porto Alegre University Hospital

**Keywords:** frontal sinus, trephination

## Abstract

The complex anatomy of the frontoethmoidal recess, as well as its anatomical relationship with the vital adjacent structures in the region explain the reason for considerable surgical care to protect these structures and minimize complications related to healing. Trephination is an accepted procedure to access the frontal sinus.

**Aim:**

Discuss the best location for performing frontal sinus trephination.

**Methods:**

Measuring sinus frontal depth at 3 points equidistant to the midline (crista galli) through the axial tomographic sections.

**Results:**

We measured 138 frontal sinus (69 patients). Frontal sinus depth at 0,5 cm was statistically larger than 1 cm and 1,5 cm, as well as the 1 cm trephine point was significantly larger than 1,5 cm (12,22±4,25 vs 11,78±4,65 p<0,05; 12,22±4,25 vs 10,78±5,98 p<0,001; 11,78±4,65 vs 10,78±5,98 p<0,05). The trephine set used (maximum depth of penetration of 0,7 cm) is safe to be applied in approximately 80% of the patients.

**Conclusion:**

Analizing the results, the trephination may be performed at variable points of the frontalsinus, but the distance of 1 cm from midline appears to be safer and shows better aestethic results.

## INTRODUCTION

Historically speaking, inflammatory diseases of the frontal sinus have been managed surgically through external approaches. Among these procedures, the frontal sinus trephination was first described by Runge in 1750^1^. In recent decades, functional endoscopic sinus surgery (FESS) has been accepted as the procedure of choice to treat chronic sinusitis^2^, ^3^. Despite the progress of endoscopic surgery, the frontal sinus remains a challenge. The complex anatomy of the fronto-ethmoid recess, as well as its anatomic relation with vital structures, explain the reason for the considerable care that has to be taken during the procedure in order to preserve these structures and minimize complications related to the healing process^4^.

Frontal sinus trephination was a procedure initially developed for the purging of complicated acute processes involving this sinus. With the development of proper and safer surgical instruments, this procedure has become useful also in the treatment of chronic cases^5^, ^6^. Its use was reinforced by the discovery that most frontal sinus diseases are a consequence of alterations in the anterior ethmoid sinus^7^. Moreover, the flow of saline solution through the fronto-ethmoid process, made possible through the trephination can be extremely useful for sinus cleaning, accurate location of the ostium and collection of material for culture^8^.

**Figure 1:**
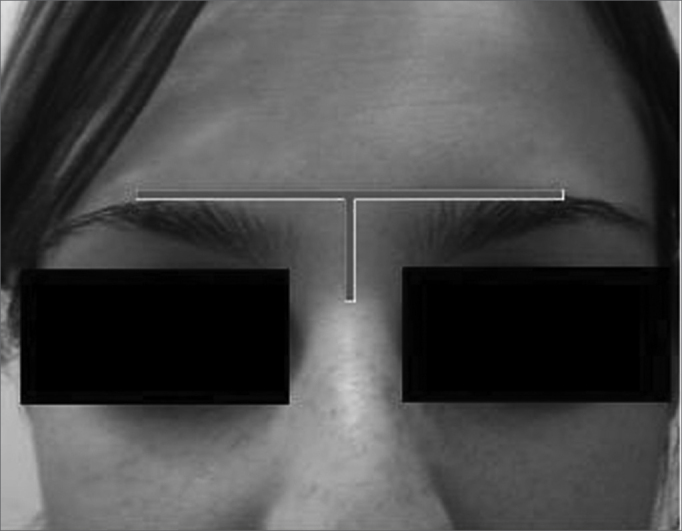
Trephination site on the frontal sinus: at the eyebrow level, 10mm from the midline.

By the technique described and accepted, the trephination is performed at the level of the eyebrows, 10mm away from a midline imaginary line between the orbits^1^ (Figure 1).

Our goal with this study is to discuss the best site to perform frontal sinus trephination, through measuring the sinus depth in 3 points - equidistant from the midline (crista galli) in axial tomographic views.

## METHODS

**Figure 2:**
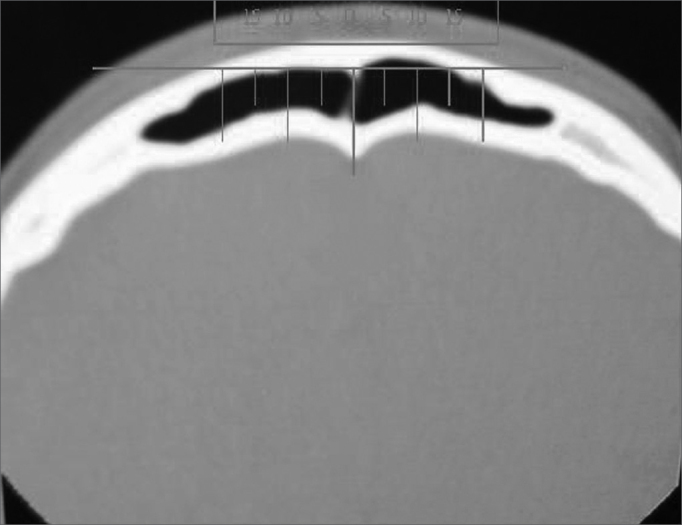
CT-scan axial view with measurements at 5, 10 and 15mm in relation to the midline (crista galli).

In a historic cohort study with cross-sections, we assessed 69 CT scans of paranasal sinuses of patients seen in the department of otorhinolaryngology of the University Hospital of Porto Alegre, corresponding to a total of 138 frontal sinuses. The CT scans were analyzed in the axial plane, and we chose the first cross section after the end of the orbit content in the cranio-caudal direction. The midline was defined by a straight line passing through the crista galli. From this point, measures with rulers were taken at 5, 10 and 15 mm from each side, checking the distance between the anterior portion of the external plate and the anterior portion of the frontal sinus posterior surface (frontal sinus depth, in mm) (Figure 2).

Patients below 2 years of age were excluded from the study.

The data was analyzed in the SPSS software, through the T Student test and Post Hoc test, in order to determine the difference between the frontal sinus depth at 5, 10 and 15mm from the midline.

## RESULTS

Of the 69 patients, 51.7% were males.

Men presented a frontal sinus significantly larger in the distances of 5 and 10mm when compared to women (p<0.001), but there was no statistical difference in the depth of the sinus at 15mm. The frontal sinus depth measured at 5mm of the midline was significantly larger than that at 10 and 15mm, just as the measure at 10mm was significantly higher when compared to that at 15mm (12.22mm ± 4.35mm vs. 11.78mm ± 4,65mm p<0.05; 12.22mm±4.35mm vs. 10.78mm±5.98mm p<0.001; 11.78mm±4.65mm vs. 10.78mm±5.98mm (p<0.05). Figure 3 shows these results.

**Chart 1:**
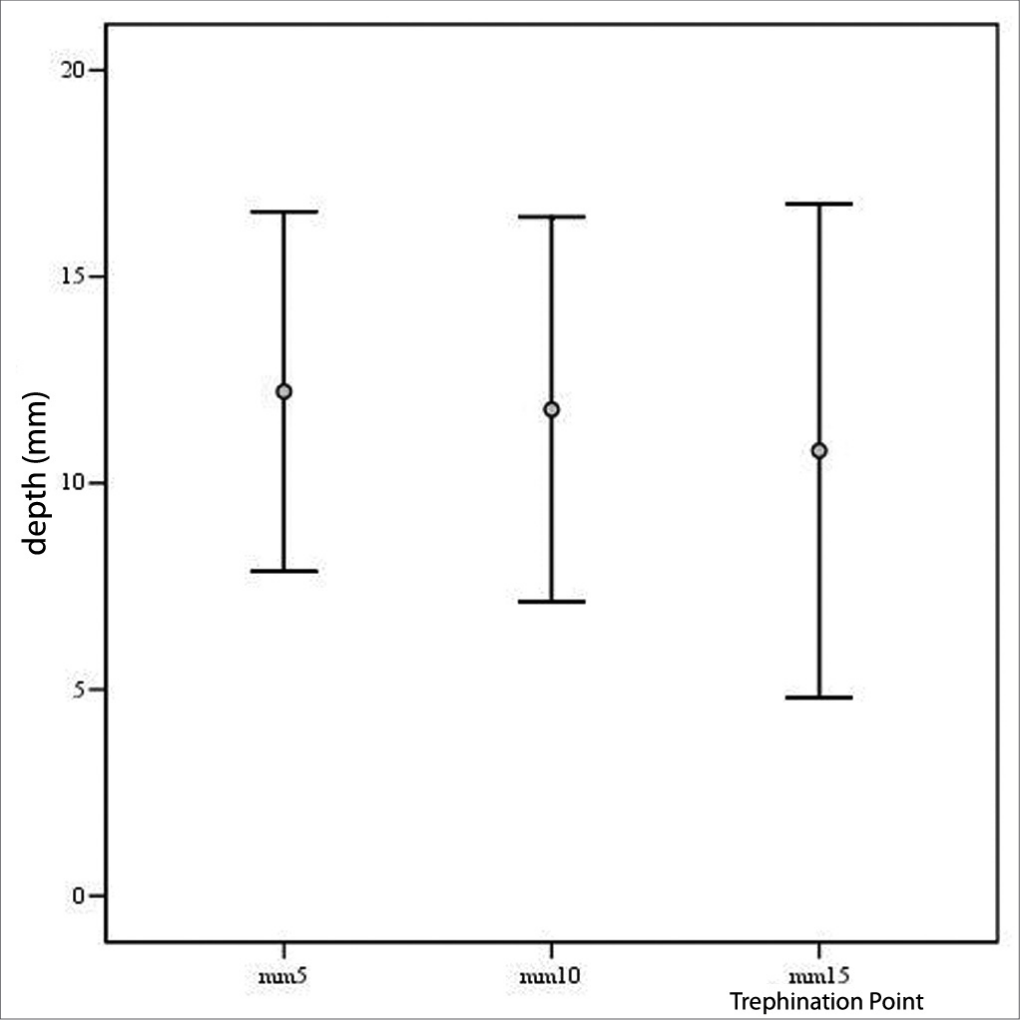
Chart showing the frontal sinus depth at each of the trephination points (mean and standard deviation).

## DISCUSSION

This greater possibility for seeing the nasal cavities and the paranasal sinuses, initially through microscopy and later with endoscopes of different angles, has marked a new expansion phase of the surgical techniques used to treat nasosinusal diseases. Regarding the frontal sinuses, its complex and varied anatomical relations with the nasal cavity and the complications accruing from its excessive manipulation have generated a number of discussions about its best approach. In these grounds, combined approaches to the frontal sinuses, through different trephination techniques have become more popular in recent years. Besides helping to identify the true fronto-ethmoidal route, such approach allows the drainage of secretions, without the need to manipulate and eventually injuring the frontal sinus ostium.

Even with the use of specific instruments that allow minimum access with depth control (Xomed®, Microfrance®), it is always necessary to measure the distance between the anterior and posterior walls of the frontal sinus in axial or sagittal view CT scan slices, in order to avoid complications during the procedure. In the literature, which includes publications from the last century, the trephination site is defined as the point 10mm away from the imaginary midline that goes through the crista galli, at the height of the orbit cranial border^1^. In our study, the sinus depth when measured at 5, 10 and 15mm away from the mid line (crista galli) showed statistically significant differences, showing that the closer the proximity to the midline, the deeper it was.

Despite all of this, trephination at 5mm from the midline must be carefully performed, since it is not always that the inter-sinus septum is located exactly in the midline. This means that, if one chooses the trephination point at 5mm from the midline, one runs the risk of performing crossed trephinations, that is, we may wish to penetrate the sinus in one side, and end up penetrating the contralateral side. Moreover, the scar may be undesirable from the cosmetic stand point, while in other points it may be hidden by the eyebrow. On the other end, the trephination point 15mm away may not be feasible on patients with sinus hypotrophy, however if the tomographic distance is properly measured, this risk is bypassed.

Statistical analysis has shown that approximately 80% of frontal sinuses depth measures are safe for trephination purposes in at least one point, using a system that requires a depth of at least 7mm in order to be called safe. Supraorbital ethmoidal cells represent another possible justification for a frontal sinus trephination at different points, as long as the image study shows that there is enough depth for such procedure

## CONCLUSION

Frontal sinus trephination is a very useful procedure for nasosinusal endoscopic surgeries. Preoperative detailed analysis of axial CT-scans or the use of computerized navigation systems are mandatory for this end and allow the measurement of frontal sinus depth at the point where trephination will be carried out.

Although different trephination points may be viable, the usual distance of 10mm from the midline proved to add more advantages.
